# Case Report: Role of numerical simulations in the management of acute aortic syndromes

**DOI:** 10.3389/fcvm.2024.1309840

**Published:** 2024-03-06

**Authors:** Antonio Rizza, Vincenzo Castiglione, Katia Capellini, Cataldo Palmieri, Emanuele Gasparotti, Sergio Berti, Simona Celi

**Affiliations:** ^1^ U.O.C. Cardiologia Diagnostica e Interventistica, Fondazione Toscana Gabriele Monasterio, Massa, Italy; ^2^ U.O.C. Cardiologia e Medicina Cardiovascolare, Fondazione Toscana Gabriele Monasterio, Pisa, Italy; ^3^ Health Science Interdisciplinary Center, Scuola Superiore Sant’Anna, Pisa, Italy; ^4^ BioCardioLab, UOC Bioingegneria, Fondazione Toscana Gabriele Monasterio, Massa, Italy

**Keywords:** penetrating aortic ulcer, acute aortic syndrome, aortic endograft, numerical simulations, computational fluid dynamics

## Abstract

Penetrating aortic ulcer (PAU) represents a subset of acute aortic syndromes characterized by high rupture risk and management challenges, particularly in elderly patients with significant comorbidities. This case report showcases a 75-year-old patient with a history of coronary artery bypass graft (CABG) and with multiple PAUs involving the aortic arch, deemed unfit for conventional open surgery. A branched aortic endograft with a pre-cannulated side component for the left subclavian artery (LSA) was employed to preserve the patency of the previous CABG. Two computational fluid dynamics (CFD) simulations and a morphological analysis were performed on the pre- and post-intervention aortic configurations to evaluate changes in flow rate and pressure drop at LSA level and differences in the lumen size. The results revealed a decrease in the flow rate equal to 2.38% after the intervention and an increase in pressure drop of 4.48 mmHg, while the maximum differences in LSA cross-sectional areas and diameters were 1.49 cm^2^ and 0.64 cm, respectively. Minimal alteration in LSA blood flow due to the chosen intervention approach confirmed the effectiveness of the selected unibody design endograft with LSA preservation, ensuring myocardial perfusion. Therefore, CFD simulations demonstrate to be a powerful tool to evaluate the hemodynamic consequences of interventions by accurately estimating the main fluid dynamic parameters.

## Introduction

Penetrating aortic ulcer (PAU) is a distinct acute aortic syndrome (AAS) characterized by erosion of the tunica intima usually due to atherosclerosis, extending deeper in the tunica media. The natural progression of PAU involves the development of medial hematoma, dissection, adventitial false aneurysm, and, potentially, transmural rupture (1, 2). The risk of rupture in PAU is significant, with rates of up to 40% (3). PAU primarily affects older individuals with a history of hypertension and smoking, particularly those over 70 years old with extensive and diffuse atherosclerotic disease (3). Although PAUs are commonly found in the descending aorta (∼90% of cases), a substantial number can also occur in the aortic arch (4).

Despite the increasing incidence of AAS, including PAU, there is a dearth of knowledge regarding optimal management strategies, and randomized trials evaluating treatment options are lacking. Current guidelines from Europe and the United States suggest that surgical intervention should be considered for lesions involving the ascending aorta (1, 2). However, the risks associated with open surgery, particularly in older patients with significant comorbidities, make endovascular approaches more attractive (5).

Numerical simulations have proven to be effective in analysing the aorta for both wall stress behaviour and fluid dynamics patterns (6, 7). In particular, computational fluid dynamics (CFD) simulations are widely adopted to investigate fluid dynamics of healthy and pathological aorta (8, 9), as well as to analyse the effects of medical devices and to predict potential negative outcomes of specific treatment procedure (10, 11).

In a previous publication (12), we presented a case of a patient with multiple PAUs and a history of coronary artery bypass graft (CABG), where we introduced an innovative solution by employing a branched aortic endograft to manage lesions affecting the aortic arch just below the left subclavian artery (LSA) to preserve the patency of the CABG. Expanding on this valuable evidence, we now propose, for the first time, the role of numerical simulations in the management of complex interventions for AAS cases, with the aim of enhancing the safety and efficacy of such procedures.

## Case presentation

A 75-year-old man was found to have multiple PAUs involving the aortic arch and descending aorta incidentally during computed tomography (CT) for prostate gland cancer staging. The patient had a history of non-ST segment elevation myocardial infarction two years ago, which was treated with CABG using the left internal mammary artery (LIMA) on the left anterior descending (LAD) artery and a saphenous vein graft on the right coronary artery. Additionally, he had a history of paroxysmal atrial fibrillation, chronic kidney disease, and previous lymphoma treated with chemotherapy and radiotherapy.

The initial work-up included a transthoracic echocardiogram, which revealed a normal left ventricular ejection fraction and moderate aortic regurgitation. The CT scan showed the largest PAU (>20 mm) located in the dorsal aortic isthmus, just below the origin of the LSA. The aortic root, ascending aorta, and aortic arch were slightly enlarged. Due to the patient's high risk for rupture and a EuroSCORE II of 20.62%, the Heart Team determined that the patient was not eligible for redo surgery.

Considering the challenging anatomy with a short landing zone and the need to preserve the LSA for blood flow from LIMA to LAD, an endograft with a pre-cannulated side component for the LSA was selected for the endovascular repair. The procedure was performed under deep sedation. Access was gained through the right radial artery and the left common femoral artery. The PAU was localized using angiography. The endograft was then advanced into the aortic arch and positioned correctly. Two endoprostheses were deployed to cover the PAUs, and no endoleaks were observed during the procedure. A follow-up CT angiogram performed five days later confirmed the proper positioning of the endografts, exclusion of all PAUs, patency of the LSA and CABG, and absence of endoleaks. The patient was discharged on the sixth postoperative day, and the three-month follow-up was uneventful.

### Technical aspects: fluid dynamics numerical simulations

In this study, a computational fluid dynamic analysis was carried out to evaluate the impact of the intervention on the flow rate through the aortic arch and the LSA.

**Figure 1:**
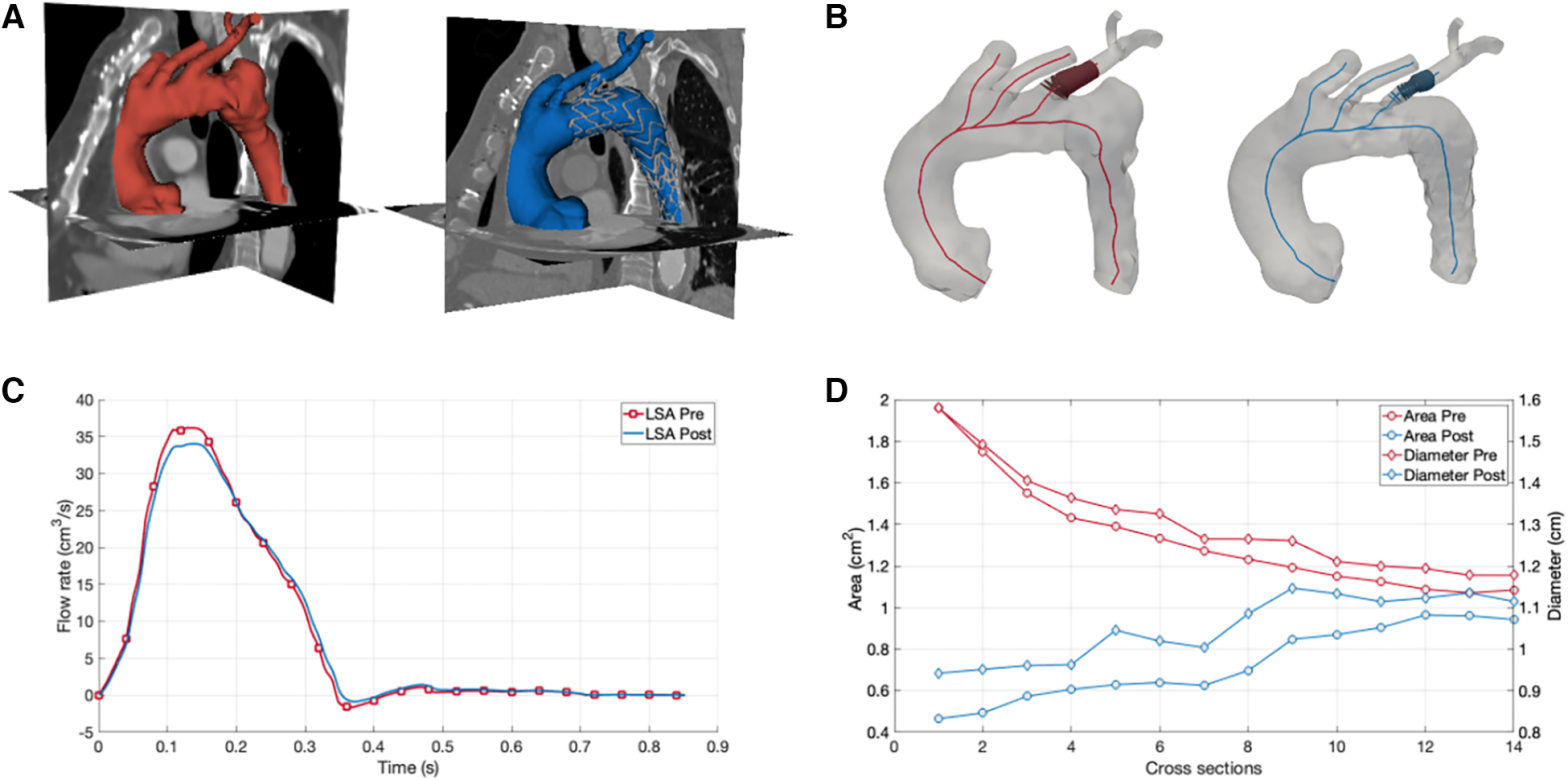
Numerical simulations of aortic arch hemodynamics and morphology for assessment of fluid dynamics changes after branched aortic endograft implantation. (**A**) Three-dimensional reconstruction of the aortic arch before (red) and after (blue) the implantation of the branched aortic endograft. (**B**) Left subclavian artery (LSA) cross sectional area and diameter before (red) and after (blue) the implantation of the branched aortic endograft. (**C**) LSA flow before (red) and after (blue) the implantation of the branched aortic endograft. (**D**) Area and diameter results for 3DPRE and 3DPOST models.

CT dataset pre- and post-intervention were retrospectively analyzed to obtain the 3D geometrical model, referred to as 3DPRE and 3DPOST, respectively. The segmentation process included the whole thoracic aorta (TA) and supra-aortic vessels (Figure 1A). The image segmentation was implemented in 3D Slicer software by using a threshold algorithm to obtain a binary mask of the anatomical regions of interest. Then, the 3D model was reconstructed by converting the binary mask in a STereo Lithography interface file.

A morphological analysis was performed to assess differences in the lumen of the LSA before and after the endoprosthesis placement. The centerline of the TA and supra-aortic vessels was computed, and cross-sections were extracted along the centerline for both the 3DPRE and 3DPOST configurations. At the level of the LSA, the maximum diameter and area were calculated for each cross-section (Figure 1B).

CFD simulations were carried out in ANSYS FLUENT software (ANSYS, Inc., Canonsburg, PA).

Regarding the computational setup, the aortic wall was assumed rigid and no-slip condition was assigned to the wall. After a mesh sensitivity analysis, the models were discretized using a polyhedral mesh, generated by using ANSYS FLUENT, with an element size averaging 0.5 mm and a total number of elements equal to 240,453. The blood was modelled as a Newtonian and incompressible fluid with a density of 1,060 kg/m^3^ and a constant viscosity of 0.0035 Pa s. Inlet boundary conditions were set using a representative aortic velocity profile, while pressure conditions were considered for the descending outlet and supra-aortic branches, matching the patient-specific pressure range (70–120 mmHg). The transient simulations were carried out over 3 cardiac cycles with a time step size of 0.004 s. The last cycle was considered for the results evaluation to ensure the stabilization of velocity and blood pressure. The same boundary conditions and computational setup parameters were applied to both the 3DPRE and 3DPOST configurations to investigate the potential morphological and fluid dynamic changes, in terms of flow rate through the LSA, due only to the intervention.

Furthermore, the pressure drop was calculated at the extremities of the LSA during the systolic peak for both configurations.

The results of the CFD simulations revealed a minimal decrease in the flow rate through the LSA after the intervention, with a percentage difference in flow volume between the 3DPRE and 3DPOST configurations during the systolic phase of only 2.38% (Figure 1C). This decrease can be attributed to a slight reduction in the lumen size of the LSA due to the insertion of the graft, as confirmed by the morphological analysis. The analysis showed differences in cross-sectional areas and diameters at the initial portion of the LSA, ranging from 0.11 to 1.49 cm^2^ and 0.04 to 0.64 cm, respectively (Figure 1). The maximum reduction in LSA lumen size after the intervention was observed at the stent graft branch at the beginning of the vessel. Furthermore, the CFD investigation revealed an increase in pressure drop in the 3DPOST configuration (4.9 mmHg) with respect to the 3DPRE configuration (0.42 mmHg).

## Discussion

The management of AAS, such as the presented case of multiple PAUs, poses significant challenges to medical teams due to the complexity and severity of the condition. Traditional treatment options, including open surgical repair, may not always be feasible or suitable for high-risk patients. In such cases, endovascular techniques have emerged as a promising alternative. However, the success of these interventions relies heavily on accurate preoperative planning and an understanding of the potential hemodynamic and morphological changes induced by the procedure. In this context, CFD simulations play a pivotal role (13, 14).

In the case at hand, the patient's medical history and the presence of multiple PAUs involving the aortic arch presented a unique challenge for the medical team. Given the patient's high surgical risk, a step-by-step approach was taken, and a unibody design endograft with a pre-cannulated side component for LSA preservation was selected. However, the short landing zone and the need to preserve blood flow to the LIMA on the LAD added complexity to the procedure.

To better understand the hemodynamic consequences of the chosen intervention, CFD simulations were performed. By reconstructing a 3D model of the TA before and after the endovascular repair, the flow rate through the LSA could be evaluated. The simulations revealed a minimal decrease in the LSA flow rate after the intervention (2.38% in the systolic phase), indicating the successful preservation of blood flow to the LIMA-LAD connection. This finding was crucial in confirming the effectiveness of the chosen unibody design endograft with LSA preservation and ensuring myocardial perfusion. Furthermore, the morphological analysis provided by the CFD simulations highlighted the changes in vessel geometry induced by the endoprosthesis placement. The reduction in LSA lumen size due to graft insertion was quantitatively assessed (up to a maximum of 1.49 cm^2^ for the cross-sectional area and 0.64 cm for diameter), shedding light on the extent of morphological changes along the vessel. This information can aid in assessing the potential risks associated with the intervention, such as increased pressure gradients or compromised blood flow, and guide post-operative monitoring and follow-up procedures. Indeed, the CFD simulations also allowed for the calculation of pressure drop between the extremities of the LSA in a non-invasive manner, which demonstrated an increase after the intervention of about 4.48 mmHg.

In this study, CFD simulations were specifically employed to model the hemodynamic consequences of aortic intervention on LSA patency within the context of a complex anatomy resulting from previous CABG. However, the use of CFD simulations for the management of AAS is extensively documented in the scientific literature, serving various purposes and at times exploring parameters different from those considered in the present study. For instance, Midulla et al. explored changes in preoperative and postoperative wall shear stress and vorticity profiles through CFD simulations in 20 patients treated for various thoracic aortic pathologies (11 aneurysms, 5 false aneurysms, 3 penetrating ulcers, and 1 traumatic aortic rupture) (10). In a single-case study, CFD simulations were employed to predict postoperative alterations in blood flow and platelet activation potential in the cervical arteries following endovascular repair in a patient with a large saccular aortic arch aneurysm (11). In one of the largest case series published to date, Polanczyk et al. employed CFD models to predict regions with lower blood velocity and shear rate, which correlated with higher blood viscosity and an increased probability of thrombus formation, in 12 patients treated for acute type B dissection (9). Collectively, these studies, along with our case, underscore the extraordinary versatility of CFD simulations in providing insights into vessel morphology and hemodynamics within the context of AAS interventional management.

This study has limitations worth noting. Firstly, it is based on a single case and on a retrospective analysis, urging caution in extending findings to diverse populations. Secondly, the use of a rigid wall assumption in aortic simulations simplifies complexities and may not fully capture real-world dynamics. Despite this, the study successfully quantified blood flow preservation through the LSA post-endovascular repair. Future work should address these limitations by analysing subjects with diverse anatomies and endograft configurations.

## Conclusions

The management of AAS, including PAUs, necessitates innovative strategies to better evaluate the different types of applied devices on the basis of their consequences on vessel morphology and hemodynamics. The reconstruction of arterial 3D model and the CFD simulations proved to be useful instruments to gain insights into vessel morphology and hemodynamics, respectively. The assessment of the impact of endovascular repair through CFD simulations confirmed the successful preservation of blood flow after the adoption of a branched aortic endograft. CFD simulations represent a patient-specific and non-invasive approach that may play a pivotal role in improving preoperative planning and optimizing outcomes by accurately predicting the impact of treatment strategy on blood flow considering the specific anatomical and physiological characteristics of each patient.

## Data Availability

The raw data supporting the conclusions of this article will be made available by the authors, without undue reservation.

## References

[1] Erbel R Aboyans V Boileau C Bossone E Bartolomeo RD Eggebrecht H 2014 ESC guidelines on the diagnosis and treatment of aortic diseases: document covering acute and chronic aortic diseases of the thoracic and abdominal aorta of the adult. The task force for the diagnosis and treatment of aortic diseases of the European Society of Cardiology (ESC). Eur Heart J. (2014) 35:2873–926. 10.1093/eurheartj/ehu281 25173340

[2] Isselbacher EM Preventza O Hamilton Black J 3rd Augoustides JG Beck AW Bolen MA, et al. 2022 ACC/AHA guideline for the diagnosis and management of aortic disease: a report of the American Heart Association/American College of Cardiology Joint Committee on clinical practice guidelines. Circulation. (2022) 146:e334–482. 10.1161/CIR.0000000000001106 36322642 PMC9876736

[3] Bischoff MS Geisbüsch P Peters AS Hyhlik-Dürr A Böckler D. Penetrating aortic ulcer: defining risks and therapeutic strategies. Herz. (2011) 36:498–504. 10.1007/s00059-011-3513-9 21887528

[4] Chou AS Ziganshin BA Charilaou P Tranquilli M Rizzo JA Elefteriades JA. Long-term behavior of aortic intramural hematomas and penetrating ulcers. J Thorac Cardiovasc Surg. Febbraio. (2016) 151:361–72, 373.e1. 10.1016/j.jtcvs.2015.09.012 26496809

[5] Rizza A Negro F Palermi S Palmieri C Murzi M Credi G Penetrating aortic ulceration treated with castor branched aortic stent graft-A case series. Int J Environ Res Public Health. (2022) 19:4809. 10.3390/ijerph19084809 35457675 PMC9033075

[6] Capellini K Gasparotti E Cella U Costa E Fanni BM Groth C A novel formulation for the study of the ascending aortic fluid dynamics with in vivo data. Med Eng Phys. Maggio. (2021) 91:68–78. 10.1016/j.medengphy.2020.09.005 33008714

[7] Celi S Gasparotti E Capellini K Bardi F Scarpolini MA Cavaliere C An image-based approach for the estimation of arterial local stiffness in vivo. Front Bioeng Biotechnol. (2023) 11:1096196. 10.3389/fbioe.2023.1096196 36793441 PMC9923115

[8] Duronio F Di Mascio A. Blood flow simulation of aneurysmatic and sane thoracic aorta using OpenFOAM CFD software. Fluids. (2023) 8:272. 10.3390/fluids8100272

[9] Polanczyk A Piechota-Polanczyk A Huk I Neumayer C Balcer J Strzelecki M. Computational fluid dynamic technique for assessment of how changing character of blood flow and different value of hct influence blood hemodynamic in dissected aorta. Diagn Basel Switz. (2021) 11:1866. 10.3390/diagnostics11101866 PMC8534802 34679564

[10] Midulla M Moreno R Negre-Salvayre A Beregi JP Haulon S Loffroy R Impact of thoracic endografting on the hemodynamics of the native aorta: pre- and postoperative assessments of wall shear stress and vorticity using computational fluid dynamics. J Endovasc Ther Off J Int Soc Endovasc Spec. (2021) 28:63–9. 10.1177/1526602820959662 33025866

[11] van Bakel TM Arthurs CJ van Herwaarden JA Moll FL Eagle KA Patel HJ A computational analysis of different endograft designs for zone 0 aortic arch repair. Eur J Cardio-Thorac Surg Off J Eur Assoc Cardio-Thorac Surg. (2018) 54:389–96. 10.1093/ejcts/ezy068 29554234

[12] Rizza A Negro F Palmieri C Clemente A Berti S. Coronary artery bypass salvage with branched aortic endograft in a patient with aortic arch ulcer. JACC Case Rep. (2022) 4:851–3. 10.1016/j.jaccas.2022.04.023 35912332 PMC9334133

[13] Biancolini ME Capellini K Costa E Groth C Celi S. Fast interactive CFD evaluation of hemodynamics assisted by RBF mesh morphing and reduced order models: the case of aTAA modelling. Int J Interact Des Manuf IJIDeM. (2020) 14:1227–38. 10.1007/s12008-020-00694-5

[14] Fanni BM Pizzuto A Santoro G Celi S. Introduction of a novel image-based and non-invasive method for the estimation of local elastic properties of great vessels. Electronics (Basel). (2022) 11:2055. 10.3390/electronics11132055

